# Effects of different types of physical exercises on gestational weight gain: a systematic review and network meta-analysis

**DOI:** 10.3389/fnut.2026.1853425

**Published:** 2026-08-24

**Authors:** Chuntian Liu, Sisi Hu, Xiaowei Shao, Zhi Lin, Miaomiao Li, Shi Qian, Xiaoxia Huang

**Affiliations:** Department of Nursing, The First Affiliated Hospital of Wenzhou Medical University, Wenzhou, Zhejiang, China

**Keywords:** gestational weight gain, network meta-analysis, physical exercise, pregnancy, systematic review

## Abstract

**Background:**

Excessive gestational weight gain (GWG) is common and associated with multiple adverse maternal and neonatal outcomes. Although physical activity during pregnancy can help control weight gain, evidence on the optimal exercise modality remains limited.

**Objective:**

To evaluate and compare the effects of different types of physical exercises on GWG using a systematic review and network meta-analysis.

**Methods:**

We systematically searched nine databases, including PubMed, Embase, Cochrane Library, CINAHL, Web of Science, Wan Fang, VIP, CNKI, and CMB from database inception to June 2025. All randomized controlled trials (RCTs) evaluating the effects of different exercise modalities on GWG were included. The quality of the included studies was assessed using the RoB 2.0 tool, and statistical analyses were performed with Stata 15.1.

**Result:**

A total of 15 studies involving 3,489 pregnant women were included, encompassing eight intervention categories: resistance exercise, aerobic exercise, combined exercise, virtual exercise, supervised exercise training, Pilates training, childbirth training, and usual care. Quality assessment indicated that five studies were at high risk of bias, four raised some concerns, and six were judged as low risk of bias. Network meta-analysis showed that no exercise modality demonstrated a statistically significant reduction in gestational weight gain compared with usual care. According to SUCRA rankings, Pilates training (84.7%), aerobic exercise (79.0%), and virtual exercise (59.1%) were ranked as the top three interventions in the primary analysis. However, sensitivity analysis excluding studies with a high risk of bias resulted in changes in treatment rankings, with aerobic exercise becoming the highest-ranked intervention.

**Conclusion:**

Current evidence does not support the superiority of any specific exercise modality for reducing gestational weight gain. Although Pilates training and aerobic exercise achieved relatively favorable rankings, these findings should be interpreted cautiously because of the limited evidence base and sensitivity of treatment rankings to study quality. Further high-quality randomized controlled trials are needed to establish the comparative effectiveness of different exercise modalities during pregnancy.

**Systematic review registration:**

https://www.crd.york.ac.uk/PROSPERO/view/CRD420251073342, identifier CRD420251073342.

## 1 Introduction

The concept of gestational weight management can be traced back to mid-1940s post-war Britain, where its primary purpose was to assess whether food rationing after World War II could provide adequate nutrition for pregnant women (1). With improvements in nutritional status, the focus of gestational weight management gradually shifted from preventing undernutrition to avoiding excessive gestational weight gain (GWG), aiming to reduce the health risks associated with GWG. According to recent literature, the incidence of GWG can reach as high as 39.4%(2). Inappropriate GWG has been linked to a variety of adverse maternal and neonatal outcomes in both the short and long term, including gestational diabetes (GDM), gestational hypertension (GH), preterm birth (PTB), macrosomia, and large-for-gestational-age (LGA) infants, among others (2, 3). Therefore, GWG has become an important public health issue that warrants urgent attention.

Physical activity (PA) is defined as any bodily movement produced by the contraction of skeletal muscles (4). A large body of evidence has demonstrated that regular and moderate PA not only contributes to controlling GWG but also reduces the risk of pregnancy- and delivery-related complications, such as cesarean section, urinary incontinence, and pelvic organ prolapse (5, 6). Accordingly, in 2005, the American College of Obstetricians and Gynecologists (ACOG) recommended that women during pregnancy and the postpartum period engage in 20 to 30 min of moderate-intensity PA on most or all days of the week (7). More recently, the 2020 World Health Organization (WHO) guidelines further emphasized that pregnant women should perform at least 150 min of moderate-intensity aerobic activity per week (8).

However, in reality, women generally have insufficient levels of PA prior to pregnancy, with only about 9%–15% of pregnant women meeting the WHO recommendations (9). In addition, most existing studies have focused on simple comparisons between a single type of exercise and either no exercise or alternative exercise modalities (10–12). Although evidence suggests that exercise during pregnancy can effectively prevent excessive GWG (6, 13), there remains a lack of conclusive evidence regarding the optimal exercise modality for gestational weight management.

Network meta-analysis (NMA), recognized as the highest level of evidence in clinical guidelines, allows the integration of both direct and indirect evidence, thereby enabling a systematic comparison of multiple interventions (14). Therefore, the present study aimed to evaluate the effects of different exercise interventions on GWG and to identify the optimal exercise modality.

## 2 Method

The present study was conducted in accordance with the Preferred Reporting Items for Systematic Reviews and Meta-Analyses (PRISMA) guidelines (15). In addition, the study protocol was registered in the PROSPERO international prospective register of systematic reviews (registration number: CRD420251073342).

### 2.1 Data sources and search strategy

We searched nine databases, including five English-language databases (PubMed, Embase, Cochrane Library, CINAHL, and Web of Science) and four Chinese-language databases (WanFang, VIP, CNKI, and CMB). The search period spanned from the inception of each database to June 2025. The detailed search strategy was provided in Supplementary Table 1.

### 2.2 Inclusion and exclusion criteria

The inclusion criteria of this study were as follows: (1) Population (P): pregnant women; (2) Interventions (I): physical activity, such as aerobic exercise, resistance exercise, or combined aerobic and resistance exercise; (3) Outcome (O): GWG; and (4) Study Design (S): randomized controlled trials (RCTs).

In addition, studies were excluded if they met any of the following criteria: (1) full text not available; (2) study protocols without complete study data; (3) unreasonable or inappropriate interventions; (4) insufficient information regarding the interventions; and (5) studies that were not randomized controlled trials (non-RCTs).

### 2.3 Literature selection and data extraction

Two authors (CTL and SSH) independently conducted the searches in the nine selected databases according to the pre-specified search strategy and recorded the titles and abstracts of studies identified during the initial screening. Another two authors (XWS and ZL) imported the initially screened studies into EndNote X20 and independently performed full-text screening based on the predefined inclusion and exclusion criteria. Any discrepancies encountered during the screening process were resolved through group discussion and consensus.

During the data extraction phase, two authors (MML and SQ) independently extracted information and data from studies that met the inclusion criteria, following the guidelines of the Cochrane Handbook for Systematic Reviews. The extracted data primarily included: (1) Basic information: first author, publication year, and study country; (2) Participants (P): inclusion criteria, sample size, and age; (3) Intervention (I): type, frequency, session duration, and total period; (4) Control (C): type, frequency, session duration, and total period; (5) Outcome (O): reported primary outcomes.

### 2.4 Risk of bias

Two authors (CTL and XXH) independently assessed the risk of bias of the included RCTs using the revised Cochrane Risk of Bias tool (RoB 2.0) (15). The risk of bias for each study across different domains, as well as the overall risk of bias, was summarized and visualized using the Robvis tool (16). Any discrepancies or uncertainties encountered during the assessment were resolved through group discussion and consensus.

### 2.5 Data analysis

The NMA was conducted using Stata 15.1 and its network and network graphs packages (17, 18). The outcome of interest was GWG, a continuous outcome; therefore, treatment effects were expressed as mean differences (MDs) with 95% confidence intervals (CIs) (19).

A network diagram including nodes and connecting lines was used to illustrate the relationships among different interventions, where the thickness of the lines represents the number of included studies and the size of the nodes corresponds to the sample size. When closed loops were present in the network, the node-splitting method was applied to assess inconsistency between direct and indirect evidence. A *P*-value ≥ 0.05 indicated consistency within the loop (20), whereas *P* < 0.05 suggested potential intransitivity, warranting further exploration of possible effect modifiers (21). In addition, a design-by-treatment model was used to evaluate global consistency across the entire network (22). Between-study heterogeneity was assessed using the between-study variance (τ^2^) derived from the random-effects network meta-analysis. Larger values of τ^2^ indicate greater heterogeneity across the included studies.

In presenting the results of the NMA, all pairwise comparisons among interventions were first displayed, clearly distinguishing between direct and indirect evidence. Subsequently, the efficacy of each intervention was ranked using the Surface Under the Cumulative Ranking Curve (SUCRA), which ranges from 0 to 100%, with higher values indicating better treatment effects (22). Finally, potential publication bias and small-study effects were assessed using a comparison-adjusted funnel plot, which is recommended for network meta-analysis. Effect estimates were centered at the comparison-specific pooled effects to account for differences across treatment comparisons. Visual inspection of funnel plot symmetry was used to evaluate the presence of potential publication bias. The certainty of evidence for the key comparisons was assessed using the Grading of Recommendations Assessment, Development and Evaluation (GRADE) approach. Evidence quality was evaluated across the domains of risk of bias, inconsistency, indirectness, imprecision, and publication bias, and categorized as high, moderate, low, or very low certainty.

## 3 Result

### 3.1 Study selection

**FIGURE 1:**
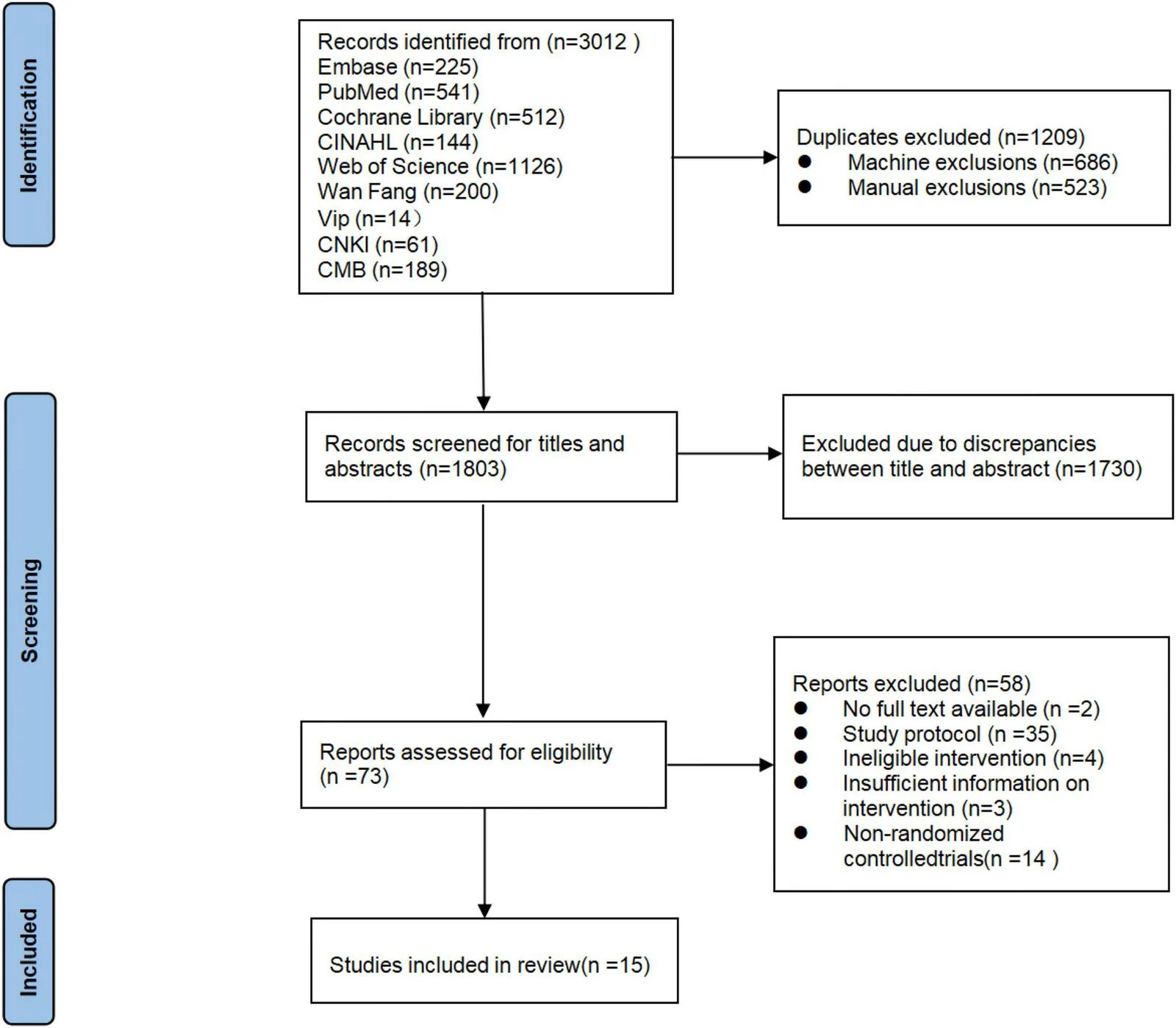
The flow chart of literature screening.

A total of 3,012 records were identified from nine databases. After removing 1,209 duplicates, 1,803 records remained. Following title and abstract screening, 1,730 records were excluded for not being relevant to the study topic. The full texts of the remaining 73 articles were then assessed, resulting in the exclusion of 58 studies, including 2 with full texts unavailable, 35 study protocols, 4 with inappropriate interventions, 3 with insufficient intervention information, and 14 non-RCTs. Ultimately, 15 studies met the inclusion criteria and were included in the analysis (10–12, 23–34). The specific screening process is illustrated in Figure 1.

### 3.2 Characteristics of included studies

As shown in Table 1, this review included 15 studies published between 2009 and 2024, with participants from seven countries: China (*n* = 4) (11, 23, 31, 34), Norway (*n* = 2) (10, 24), Spain (*n* = 5) (12, 25, 28, 29, 32), USA (*n* = 1) (26), New Zealand (*n* = 1) (27), Turkey (*n* = 1) (30), and Denmark (*n* = 1) (33), with a total sample size of 3,489. A variety of intervention types were identified, with eight main forms: aerobic exercise (*n* = 5) (11, 26, 27, 31, 34) and Combined Exercise (*n* = 5 (10, 23–25, 28) were the most common followed by resistance exercise (*n* = 2) (31, 34), virtual exercise (*n* = 1) (32), structured supervised training (*n* = 3) (12, 29, 33), Pilates (*n* = 1) (30) childbirth training (*n* = 1) (30) and usual care, which was provided in the majority of studies (*n* = 14) (10–12, 23–33). The duration of interventions varied considerably, ranging from 4 to 31 weeks (30, 32).

**TABLE 1:** Characteristics of included studies.

Study Country	Participants a. Inclusion criteria b. Sample size (intervention vs. control) c. Age (intervention vs. control)	Intervention a. Method b. Frequency c. Duration d. Intervention Period	Control a: Method b. Frequency c. Duration d. Intervention period	Outcome
Ma et al., (23) China	a. Pregnant women who regularly attend prenatal care and participate in perinatal health education, gestational age < 24 weeks, singleton primiparas, and no pregnancy-related complications or comorbidities b. 344 (129 vs. 215) c. 26.52 ± 3.00 vs. 28.26 ± 3.21	a. Combined exercise b. Exercise intensity was adjusted according to gestational age d. From 24 weeks of gestation until delivery	a. Usual care	GWG; self-reported satisfaction with mental and physical health
Haakstad, (24) Norway	a. Pregnant women who are able to read, comprehend, and speak Norwegian b. 105 (52 vs. 53) c.31.2 ± 3.7 vs. 30.3 ± 4.4	a. Combined Exercise b. At least 2 sessions/week c. Aerobic dance combined with 60-min strength training d. At least 12 weeks	a. Usual care	GWG (including proportion of women whose weight gain exceeded the recommendations of the Institute of Medicine);average skinfold thickness; postpartum weight retention rate
Ruiz et al., (25) Spain	a. Sedentary women with singleton pregnancies and no complications, who were at low risk of preterm birth and had not participated in any other trials, took part in this study b. 5–6 weeks c. 962(481 vs. 481) d. 31.9 ± 4 vs. 31.6 ± 4	a. Combined exercise b. 3 sessions/week (1 moderate-intensity aerobic + 2 resistance sessions/week) c. 50–55 min d. 9–38/39 weeks of gestation	a. Usual care	GWG; maternal and neonatal outcomes
Kong et al., (26) USA	a. Women aged 18–45 years with a singleton pregnancy; non-smokers, BMI ≥ 25 or 30 kg/m^2^, no history of chronic diseases and without GDM b. 15–40 weeks c. 37 (18 vs. 19) d. 27.4 ± 4.23 vs. 26.54 ± 3.78	a. Aerobic exercise b. 5 sessions/week c. Week 1: 50 min; Week 2: 100 min; Week 3: majority of days with 30 min/day d. At least 20 weeks	a. Usual care	Daily total steps; moderate-intensity pace; GWG; pregnancy complications and neonatal outcomes
Seneviratne et al., (27) New Zealand	a. Pregnant women with a BMI ≥ 25 kg/m^2^ and a singleton pregnancy b. < 20 weeks c. 75 (37 vs. 38) d. 18–40	a. Aerobic exercise b. 3–5 sessions/week c. 13–30 min d. 16 weeks	a. Usual care	Offspring birth weight; pre-specified maternal and perinatal parameters
Garnæs et al., (10) Norway	a. BMI ≥ 28 kg/m^2^,age > 18 years, singleton live pregnancy, able to attend assessment and training at St. Olavs Hospital b. < 18 weeks c. 91 (46 vs. 45) d. 31.3 ± 3.8 vs. 31.4 ± 4.7 vs.	a. Combined Exercise b. 3 sessions/week c. 60 min (35 min walking + 25 min resistance training for major muscle groups and pelvic floor muscles d. 12 weeks’ gestation – delivery	a. Usual care	GWG;GDM; blood pressure etc.,
Wang et al., (11) China	a. Women who were overweight or obese and non-smokers b. < 12 weeks c. 200(150 vs. 50) d: 32.14 ± 4.57 vs. 32.50 ± 4.91	a. Aerobic exercise b: At least 3 sessions/week c. 45–60 min d. Grouping day 3 – 36/37 weeks’ gestation	a. Usual care	Incidence of GDM; physical activity level; GWG; biochemical outcomes; maternal and perinatal outcomes etc.,
Bacchi et al., (28) Spain	a. Singleton pregnancy with no complications c. 111(49 vs. 62) d. 30.4 ± 4 vs. 31.0 ± 5	a. Combined Exercise b. 3 sessions/week c. 55–60 min d. 8–11 weeks’ gestation–38/39 weeks’ gestation	a. Usual care	GWG; birth weight; gestational age (days);maternal blood pressure; infant length; head circumference and Apgar scores
Barakat et al., (29) Spain	a. Healthy pregnant women from two medical centers b. > 8 weeks c. 456(234 vs. 222) d. 31.75 ± 4.68 vs. 31.04 ± 3.78	a. Supervised exercise training b. 3 sessions/week c. 55–60 min d. 8–10 weeks’ gestation – 38/39 weeks’ gestation	a. Usual care	GWG; OGTT and GDM etc.,
Pelaez et al., (12) Spain	a. Pregnant women with healthy, singleton, uncomplicated pregnancies, not participating in other exercise programs b. > 8 weeks c. 301(100 vs. 201) d.31.07 ± 3.19 vs. 31.54 ± 4.7	a. Supervised exercise training b. 3 sessions/week c. 60–65 min	a. Usual care	BMI categories; GWG
Aktan et al., (30) Turkey	a. Age ≤ 15 years, no pregnancy-related complications c. 63(21 vs. 21 vs. 22) d. 27.52 ± 3.88 vs. 25.85 ± 3.63 vs. 25.5 ± 4.19	a_1_. Pilates training a_2_. Childbirth training b_1_. 2 sessions/week b _2_. 1 session/week c. 1 h/session d. Weeks	a. Usual care	GWG; anxiety score; mode of delivery and other related outcomes
Zhang et al., (31) China	a. Healthy pregnant women b. > 12 weeks c. 319(102 vs. 117 vs. 100) d. 26.8 ± 3.4 vs. 27.4 ± 3.1 vs. 27.1 ± 2.9	a_1_. Aerobic exercise a_2_. Resistance exercise b _1/2_: 3–5 sessions/week c_1._ ≥ 30 min c_2._ 30 min d. > 4 months	a. Usual care	Adverse maternal and perinatal outcomes; GWG; total labor duration; cesarean delivery; postpartum hemorrhage
Silva-Jose et al., (32) Spain	a. Healthy pregnant women b. > 8 weeks c: 139(69 vs. 70) d: 33.83 ± 3.87 vs. 33.41 ± 5.91	a. Virtual exercise b. 3 sessions/week c. 55–60 min d. 30–31 weeks	a. Usual care	GWG; neonatal outcomes; birth weight
Roland et al., (33) Denmark	a. Healthy sedentary women b. < 15–40 weeks c. 178(74 vs. 70 vs.34) d. 31.1 ± 4.3 vs. 31.7 ± 4.1 vs. 32.0 ± 4.6	a. Supervised exercise training b. 3 sessions/week c. 30 min of stationary cycling and 30 min of other exercises d. 28 weeks	a. Usual care	GWG; obstetric outcomes; neonatal outcomes
Liu et al., (34) China	a. GDM patients under prenatal follow-up b. 24–30 weeks c. 108 (54 vs. 54) D:3.14 ± 2.66 vs. 30.39 ± 2.63	a. Resistance exercise b. 3 sessions/week c. 50–60 min d. At least 6 weeks	a. Aerobic exercise	Fasting and 2-h postprandial blood glucose before and after intervention; pregnancy outcomes: GWG; cardiometabolic profile

GWG, Gestational Weight Gain; BMI, Body Mass Index; GDM, Gestational diabetes mellitus.

### 3.3 Risk of bias

Among the 15 included studies, five were assessed as having a high risk of bias (12, 23, 26, 30, 31), mainly due to lack of random allocation, bias in the intervention process, missing data during follow-up, and limitations in outcome measurement methods. Four studies were rated as some concerns (10, 32–34), primarily because of lack of random allocation and bias in intervention implementation. The remaining six studies were judged to have a low risk of bias (11, 24, 25, 27–29). Detailed assessment results are presented in Supplementary Figures 1, 2.

### 3.4 Network diagram

**FIGURE 2:**
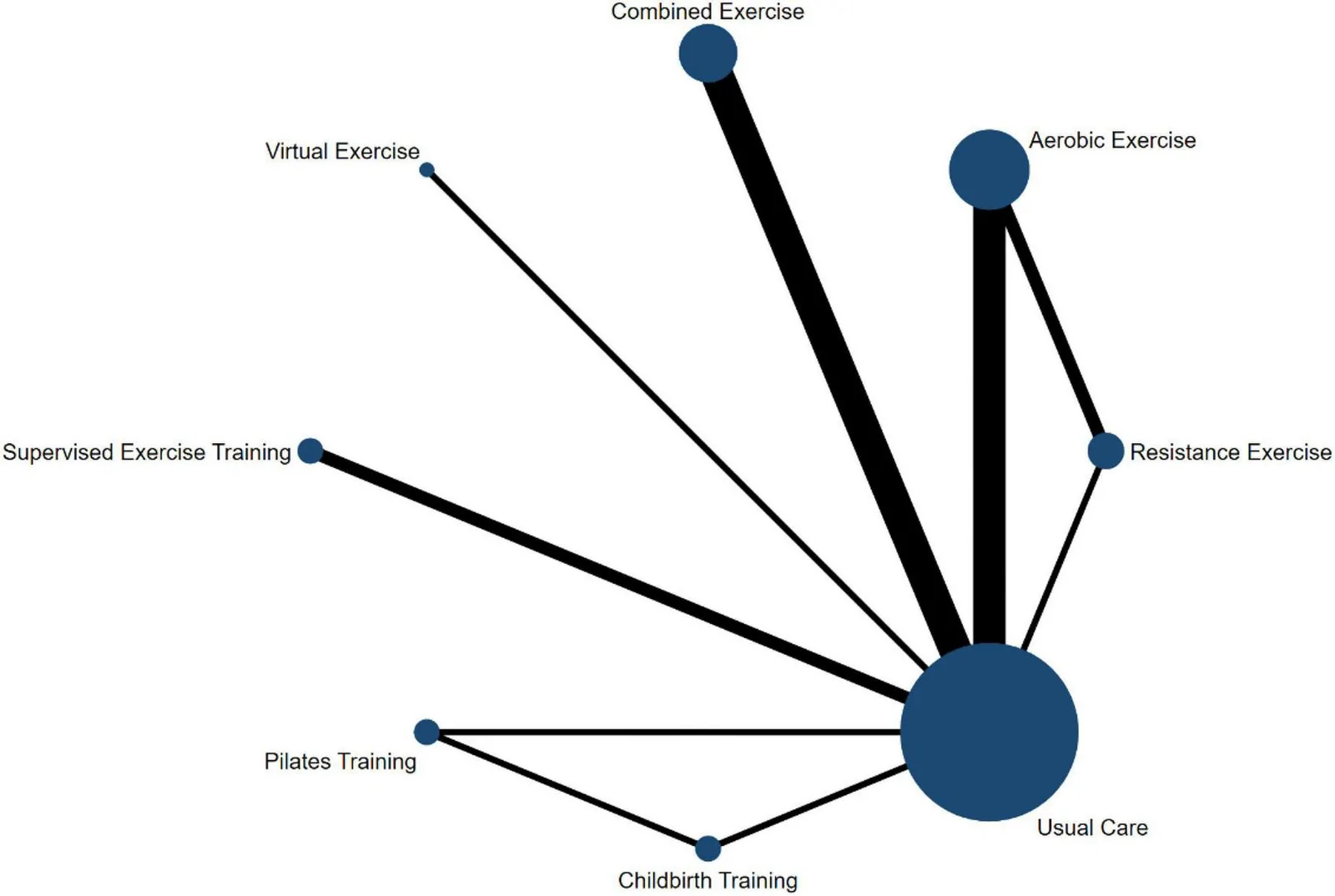
Network diagram of all interventions included in the network meta-analysis. The cumulative sample sizes are presented for descriptive purposes only and do not represent single pooled treatment groups in the network meta-analysis model.

Among the fifteen included studies, eight intervention categories were identified. The network comprised nine direct comparisons, resulting in a total of twenty-eight pairwise treatment comparisons evaluated through direct and/or indirect evidence. The most frequently reported interventions were usual care (*n* = 14), aerobic exercise (*n* = 5), and combined exercise (*n* = 5). Regarding the distribution of sample sizes, usual care had the largest cumulative sample size (*n* = 1,612), followed by combined exercise (*n* = 802), structured supervised exercise training (*n* = 408), aerobic exercise (*n* = 361), resistance exercise (*n* = 171), virtual exercise programs (*n* = 69), Pilates training (*n* = 21), and childbirth training (*n* = 21). Most interventions were connected through the usual care node, although two closed loops were present within the network. The network geometry is presented in Figure 2.

### 3.5 Consistency and heterogeneity assessment

The random-effects network meta-analysis demonstrated substantial between-study heterogeneity, with an estimated between-study variance of τ^2^ = 4.55 (τ = 2.13). Potential sources of heterogeneity included differences in participant characteristics, exercise frequency, intervention duration, and gestational age at enrollment. In the primary analysis, evidence of inconsistency was observed within the network. After excluding the five studies judged to be at high risk of bias, both global and local inconsistency tests indicated no significant inconsistency (global inconsistency test: χ^2^ = 2.01, *p* = 0.156). Detailed results are presented in Supplementary Table 2.

### 3.6 Network meta-analysis

As shown in Table 2, the network meta-analysis revealed that most pairwise comparisons were not statistically significant, with confidence intervals crossing the null value. Aerobic exercise showed a significantly higher GWG compared with usual care (MD = 2.52, 95% CI: 0.46 to 4.59). No significant differences were observed between resistance exercise and the remaining exercise interventions. These findings indicate substantial uncertainty in the relative treatment effects across interventions.

**TABLE 2:** The network meta-analysis for all interventions.

Pilates training	1.42 (−4.19, 7.02)	2.35 (−4.50, 9.20)	3.46 (−2.12, 9.05)	3.51 (−1.74, 8.76)	3.56 (−2.53, 9.66)	3.94 (−1.27, 9.15)	4.47 (−1.55, 10.49)
−1.42 (−7.02, 4.19)	Aerobic exercise	0.93 (−3.97, 5.84)	2.05 (−0.82, 4.92)	2.09 (−3.64, 7.83)	2.15 (−0.70, 4.99)	**2.52** **(0.46, 4.59)**	3.05 (−0.60, 6.70)
−2.35 (−9.20, 4.50)	−0.93 (−5.84, 3.97)	Virtual exercise	1.11 (−3.76, 5.99)	1.16 (−5.80, 8.12)	1.21 (−4.23, 6.66)	1.59 (−2.86, 6.04)	2.12 (−3.25, 7.49)
−3.46 (−9.05, 2.12)	−2.05 (−4.92, 0.82)	−1.11 (−5.99, 3.76)	Combined exercise	0.05 (−5.67, 5.76)	0.10 (−3.63, 3.83)	0.48 (−1.52, 2.47)	1.00 (−2.61, 4.62)
−3.51 (−8.76, 1.74)	−2.09 (−7.83, 3.64)	−1.16 (−8.12, 5.80)	−0.05 (−5.76, 5.67)	Childbirth training	0.05 (−6.16, 6.26)	0.43 (−4.92, 5.78)	0.96 (−5.18, 7.10)
−3.56 (−9.66, 2.53)	−2.15 (−4.99, 0.70)	−1.21 (−6.66, 4.23)	−0.10 (−3.83, 3.63)	−0.05 (−6.26, 6.16)	Resistance exercise	0.38 (−2.78, 3.53)	0.90 (−3.46, 5.26)
−3.94 (−9.15, 1.27)	−**2.52** **(−4.59, −0.46)**	−1.59 (−6.04, 2.86)	−0.48 (−2.47, 1.52)	−0.43 (−5.78, 4.92)	−0.38 (−3.53, 2.78)	Usual care	0.53 (−2.48, 3.54)
−4.47 (−10.49, 1.55)	−3.05 (−6.70, 0.60)	−2.12 (−7.49, 3.25)	−1.00 (−4.62, 2.61)	−0.96 (−7.10, 5.18)	−0.90 (−5.26, 3.46)	−0.53 (−3.54, 2.48)	Supervised exercise training

Results are expressed as mean differences (MDs) with corresponding 95% confidence intervals (CIs). Bold values indicate statistically significant differences (*p* < 0.05).

### 3.7 Probability ranking

**FIGURE 3:**
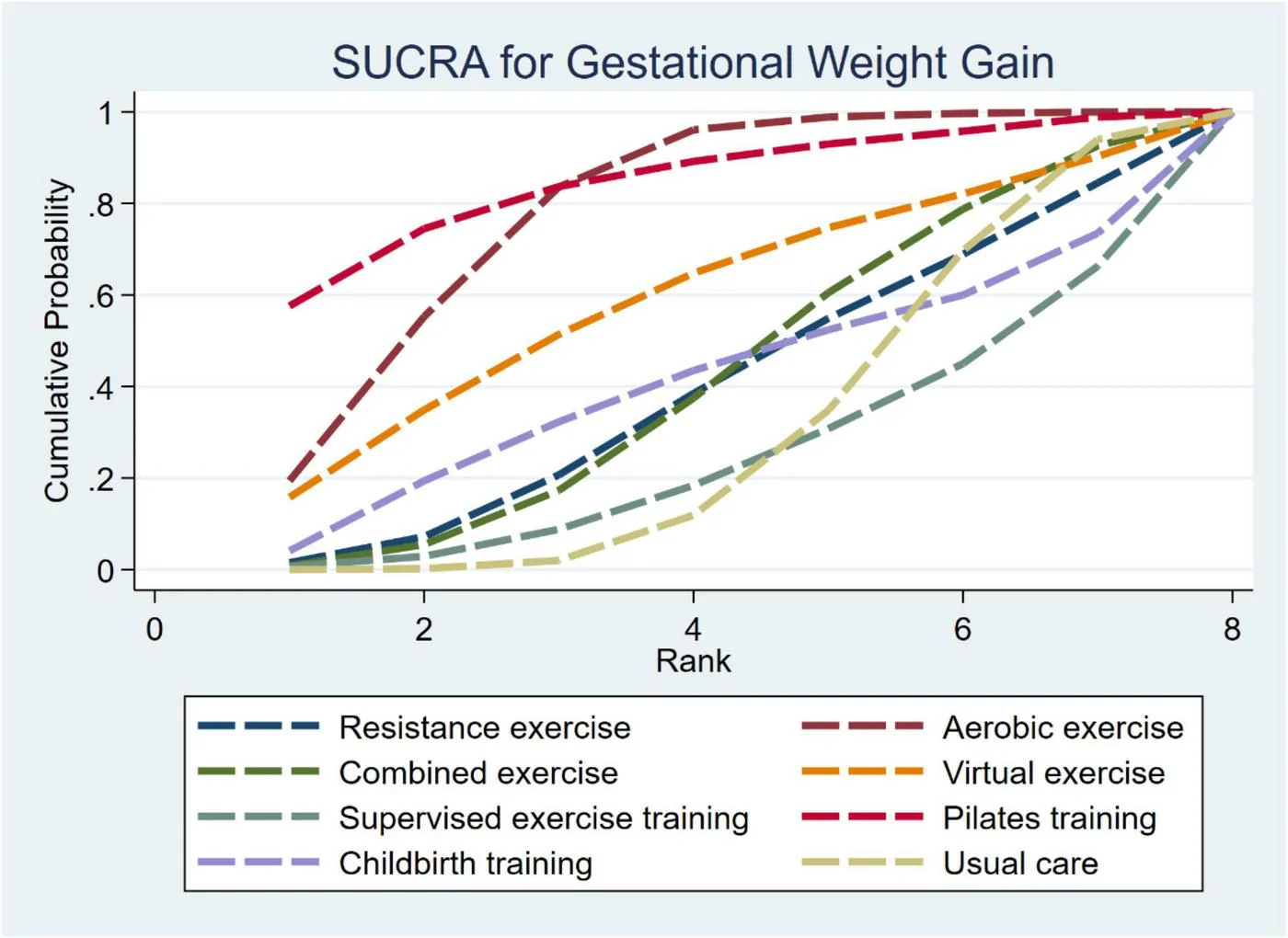
The probability ranking for all interventions.

When ranking the interventions according to SUCRA values, Pilates training (84.7%), aerobic exercise (79.0%), and virtual exercise (59.1%) were ranked as the top three interventions. The SUCRA values for the remaining interventions were combined exercise (41.9%), childbirth training (40.8%), resistance exercise (39.5%), usual care (30.3%), and supervised exercise training (24.7%). Detailed ranking results are presented in Figure 3.

### 3.8 Publication bias

The comparison-adjusted funnel plot showed that most study points were distributed around the vertical reference line. While a small degree of scatter was observed, no clear visual evidence of substantial asymmetry was identified. The comparison-adjusted funnel plot is presented in Supplementary Figure 3.

### 3.9 Sensitivity analysis

A sensitivity analysis was conducted after excluding the five studies assessed as having a high risk of bias. The node-splitting results of the sensitivity analysis are presented in Supplementary Table 3. Following exclusion, six intervention categories remained in the network. Compared with the primary analysis, the ranking of interventions changed, with aerobic exercise ranking highest, followed by virtual exercise and supervised exercise training. Notably, Pilates training, which ranked first in the primary analysis, was no longer included in the sensitivity analysis because the corresponding study was excluded due to high risk of bias. Detailed league table and SUCRA ranking results are presented in Supplementary Figure 4 and Supplementary Table 4. These findings suggest that treatment rankings were influenced by study quality and should therefore be interpreted with caution. The certainty of evidence was further assessed using the GRADE approach. For the comparison between aerobic exercise and usual care, the certainty of evidence was rated as low because of concerns regarding risk of bias, indirectness, and imprecision. Detailed results of the GRADE assessment are presented in Supplementary Table 5.

## 4 Discussions

This network meta-analysis evaluated the effects of different exercise modalities on GWG and provides comparative evidence for exercise-based gestational weight management. Overall, no exercise modality showed a statistically significant reduction in GWG compared with usual care. According to SUCRA values in the primary analysis, Pilates training ranked highest, followed by aerobic exercise and virtual exercise. However, these ranking results should be interpreted cautiously because the highest-ranked intervention was informed by a limited evidence base and was no longer available in the sensitivity analysis after excluding studies with a high risk of bias. In the sensitivity analysis, aerobic exercise ranked highest, followed by virtual exercise and supervised exercise training, suggesting that treatment rankings were influenced by study quality and the underlying evidence base.

Several factors may explain why no exercise modality demonstrated a statistically significant reduction in GWG compared with usual care in the present NMA. First, GWG is a complex outcome influenced by multiple factors beyond physical activity, including maternal dietary intake, pre-pregnancy BMI, metabolic status, and gestational age. As a result, the independent contribution of exercise may be difficult to isolate. Second, considerable variation existed across the included studies regarding exercise type, intensity, frequency, duration, and intervention delivery, which may have reduced the ability to detect differences between exercise modalities. In addition, participant adherence may have influenced intervention effectiveness. Barakat et al. (35). reported that successful exercise interventions were generally characterized by higher adherence and lower attrition rates, highlighting the importance of sustained participation during pregnancy. Furthermore, no consistent relationship between exercise dose and GWG reduction has been established (35). Current clinical guidelines recommend both aerobic and strength-conditioning exercises during pregnancy but acknowledge that the optimal exercise modality, frequency, and intensity remain uncertain (36). Therefore, the lack of significant differences observed in the present study may reflect the multifactorial nature of GWG and the heterogeneity of exercise interventions rather than the absence of potential benefits from physical activity.

In the primary analysis, Pilates training achieved the highest SUCRA ranking among all exercise modalities. However, this finding should be interpreted with considerable caution. The ranking was informed by only a single randomized controlled trial involving 63 participants, resulting in a limited evidence base and substantial uncertainty regarding the estimated treatment effect. Although Pilates training ranked highest in the treatment hierarchy, no statistically significant difference was observed between Pilates training and usual care. Therefore, the ranking result alone should not be interpreted as definitive evidence of superiority. Furthermore, Pilates training was no longer represented in the sensitivity analysis after exclusion of studies with a high risk of bias, indicating that its ranking was highly dependent on the inclusion of a single study. This finding suggests that the treatment hierarchy was sensitive to study quality and that the apparent advantage of Pilates training may not be robust. Similar concerns have been raised in network meta-analysis methodology, where treatment rankings based on sparse evidence may be unstable and should be interpreted cautiously, particularly when informed by a limited number of studies and participants (22). Importantly, SUCRA values reflect the probability that an intervention ranks among the best treatments rather than providing direct evidence of clinical superiority (22). Consequently, a high SUCRA value does not necessarily indicate a statistically significant or clinically meaningful advantage over competing interventions. Therefore, the apparent superiority of Pilates training should be regarded as exploratory rather than conclusive. Additional high-quality randomized controlled trials with larger sample sizes and direct head-to-head comparisons are required before firm conclusions can be drawn regarding the effectiveness of Pilates training for controlling gestational weight gain.

Although no exercise modality demonstrated statistically significant superiority over usual care, aerobic exercise showed relatively consistent performance across analyses. In the primary analysis, aerobic exercise ranked second according to SUCRA values, and it became the highest-ranked intervention in the sensitivity analysis after exclusion of studies with a high risk of bias. This relative stability may suggest that aerobic exercise is supported by a comparatively more consistent evidence base than some other exercise modalities evaluated in the present network.

Our findings are broadly consistent with previous evidence indicating that physical activity interventions can contribute to gestational weight management and reduce the risk of excessive gestational weight gain. Recent large-scale meta-analyses have identified physical activity as an important component of antenatal weight management strategies and have highlighted the role of exercise in improving maternal outcomes. Nevertheless, despite its relatively favorable ranking, no statistically significant differences were observed between aerobic exercise and usual care in the present NMA. Therefore, aerobic exercise should not be interpreted as definitively superior to other exercise modalities, and further direct comparative trials are required to establish whether specific exercise types provide greater benefits for gestational weight management.

Although no exercise modality demonstrated statistically significant superiority over usual care, aerobic exercise showed relatively consistent performance across analyses. In the primary analysis, aerobic exercise ranked second according to SUCRA values and became the highest-ranked intervention in the sensitivity analysis after exclusion of studies with a high risk of bias. This finding suggests that the ranking of aerobic exercise was less influenced by study quality than that of some other exercise modalities. The relatively stable performance of aerobic exercise may be related to the fact that it is one of the most extensively studied and commonly recommended forms of exercise during pregnancy. Previous evidence has suggested that aerobic exercise training can contribute to the control of maternal weight gain during pregnancy (37). Nevertheless, despite its favorable ranking in the present NMA, no statistically significant differences were observed between aerobic exercise and usual care. Therefore, the present findings should not be interpreted as evidence that aerobic exercise is superior to other exercise modalities. Rather, aerobic exercise may represent a reasonable and evidence-supported option for gestational weight management, while further direct comparative trials are needed to determine the comparative effectiveness of different exercise types.

Several limitations of this study should be acknowledged. First, some exercise modalities were informed by a limited number of studies and participants. In particular, the ranking of Pilates training was based on a single randomized controlled trial involving only 63 participants, which increases uncertainty regarding the estimated treatment effects. Second, several treatment comparisons relied primarily on indirect evidence within the network, which may reduce the certainty of comparative estimates. Third, the sensitivity analysis demonstrated changes in treatment rankings after exclusion of studies with a high risk of bias, indicating that the results were influenced by study quality and should therefore be interpreted with caution. Finally, variations in exercise intensity, frequency, duration, and intervention delivery across studies may have contributed to clinical heterogeneity. Future research should prioritize large-scale, high-quality randomized controlled trials directly comparing different exercise modalities using standardized intervention protocols. Such studies would help strengthen the evidence base and provide more definitive conclusions regarding the comparative effectiveness of exercise interventions for gestational weight management.

## 5 Conclusion

Current evidence does not support the superiority of any specific exercise modality for reducing gestational weight gain. While Pilates training and aerobic exercise achieved relatively favorable rankings in the present network meta-analysis, these findings should be interpreted cautiously because of the limited evidence base and changes observed in sensitivity analyses. Further large-scale, high-quality randomized controlled trials are needed to provide more robust evidence regarding the comparative effectiveness of different exercise modalities during pregnancy.

## Data Availability

The raw data supporting the conclusions of this article will be made available by the corresponding author upon request
