# Supplementary material for: Effects of different types of physical exercises on gestational weight gain: a systematic review and network meta-analysis
Source: Front Nutr. 2026 Aug 24;13:1853425. doi: 10.3389/fnut.2026.1853425 (PMC13547060; doi:10.3389/fnut.2026.1853425)
Supplement: Supplementary file 1 [file Supplementary_file_1.docx]

Supplementary Table 1：Searching Strategy

Supplementary Figure 1-2：Risk of Bias Assessment of Included Studies

**Supplementary Table 2:**Results of node-splitting analysis

**Supplementary Table 3:**Node-splitting results in sensitivity analysis

**Supplementary Table 4:Network meta-analysis league table after exclusion of studies at high risk of bias**

**Supplementary Figure 3:**Publication Bias for Gestational Weight Gain

Supplementary Figure 4:SUCRA ranking after exclusion of studies at high risk of bias

Supplementary Table 5:GRADE Assessment of the Comparison Between Aerobic Exercise and Usual Care

| Supplementary Table 1：Searching Strategy | | | |
| --- | --- | --- | --- |
| Database | Date Searched | Search Strategy | Number of References Retrieved |
| Pubmed | 16/6/2025 | #1"Pregnant People"[MeSH Terms] | 16804 |
|  |  | #2"(((((((("Pregnant People"[Mesh]) OR (pregnan*[Title/Abstract])) OR (People, Pregnant[Title/Abstract])) OR (Pregnant Peoples[Title/Abstract])) OR (Pregnant Person[Title/Abstract])) OR (Pregnant Woman[Title/Abstract])) OR (Woman, Pregnant[Title/Abstract])) OR (Women, Pregnant[Title/Abstract])) OR (Pregnant Women[Title/Abstract])"[Title/Abstract] | 673559 |
|  |  | #3 #1OR#2 | 673559 |
|  |  | #4" Exercise"[MeSH Terms] | 272682 |
|  |  | #5"(((((("Exercise"[Mesh]) OR (Exercis*[Title/Abstract])) OR (physical activit*[Title/Abstract])) OR (exercise training*[Title/Abstract])) OR (aerobic exercis*[Title/Abstract])) OR (isometric exercis*[Title/Abstract])) OR (acute exercis*[Title/Abstract]) | 635413 |
|  |  | #6 #4 OR #5 | 635413 |
|  |  | #7 "Gestational Weight Gain"[Mesh] | 1758 |
|  |  | #8"((((((("Gestational Weight Gain"[Mesh]) OR (Weight Gain, Gestational[Title/Abstract])) OR (Maternal Weight Gain[Title/Abstract])) OR (Weight Gain, Maternal[Title/Abstract])) OR (Pregnancy Weight Gain[Title/Abstract])) OR (Weight Gain, Pregnancy[Title/Abstract])) OR (Postpartum Weight Retention[Title/Abstract])) OR (Weight Retention, Postpartum[Title/Abstract]) | 4109 |
|  |  | #9#7OR#8 |  |
|  |  | #10 Randomized Controlled Trial" [Publication Type] | 641141 |
|  |  | #11 (((("Randomized Controlled Trial" [Publication Type]) OR (random*[Title/Abstract])) OR (randomized controlled trial[Title/Abstract])) OR (blind[Title/Abstract])) OR (double[Title/Abstract]) | 2268420 |
|  |  | # 12 #10 OR #11 | 2268420 |
|  |  | #13 #3AND #6 AND#9AND#12 | 221 |
| Embase | 16/6/2025 | #1'pregnant people'/exp OR 'pregnant people' OR (pregnant AND people) OR pregnan OR 'people, pregnant' OR (people, AND pregnant) OR 'pregnant peoples' OR (pregnant AND peoples) OR 'pregnant person'/exp OR 'pregnant person' OR (pregnant AND person) OR 'pregnant woman'/exp OR 'pregnant woman' OR (pregnant AND ('woman'/exp OR woman)) OR 'woman, pregnant' OR (('woman,'/exp OR woman,) AND pregnant) OR 'women, pregnant' OR (('women,'/exp OR women,) AND pregnant) OR 'pregnant women'/exp OR 'pregnant women' OR (pregnant AND ('women'/exp OR women)) | 346041 |
|  |  | #2 ((((('exercise'/exp OR exercise OR exercis* OR physical) AND activit* OR 'exercise'/exp OR exercise) AND training* OR aerobic) AND exercis* OR isometric) AND exercis* OR acute) AND exercis* | 267057 |
|  |  | #3'gestational weight gain'/exp OR 'gestational weight gain' OR (gestational AND ('weight'/exp OR weight) AND ('gain'/exp OR gain)) OR 'weight gain, gestational' OR (('weight'/exp OR weight) AND gain, AND gestational) OR 'maternal weight gain'/exp OR 'maternal weight gain' OR (('maternal'/exp OR maternal) AND ('weight'/exp OR weight) AND ('gain'/exp OR gain)) OR 'weight gain, maternal' OR (('weight'/exp OR weight) AND gain, AND ('maternal'/exp OR maternal)) OR 'pregnancy weight gain'/exp OR 'pregnancy weight gain' OR (('pregnancy'/exp OR pregnancy) AND ('weight'/exp OR weight) AND ('gain'/exp OR gain)) OR 'weight gain, pregnancy' OR (('weight'/exp OR weight) AND gain, AND ('pregnancy'/exp OR pregnancy)) OR 'postpartum weight retention'/exp OR 'postpartum weight retention' OR (('postpartum'/exp OR postpartum) AND ('weight'/exp OR weight) AND ('retention'/exp OR retention)) OR 'weight retention, postpartum' OR (('weight'/exp OR weight) AND retention, AND ('postpartum'/exp OR postpartum)) | 33446 |
|  |  | #4 #1AND #2 AND#3 | 225 |
|  |  |  |  |
| Web Of Science | 16/6/2025 | #1TS=(Pregnant People) | 18914 |
|  |  | #2AB=(Pregnant People OR pregnan OR People, Pregnant OR Pregnant Peoples OR Pregnant Person OR Pregnant Woman OR Woman, Pregnant OR Women, Pregnant OR Pregnant Women) | 189121 |
|  |  | #3 #1OR#2 | 192972 |
|  |  | #4TS=(Exercise) | 985414 |
|  |  | #5AB=(Exercise OR Exercis* OR physical activit* OR exercise training* OR aerobic exercis* OR isometric exercis* OR acute exercis*) | 929482 |
|  |  | #6 #4OR#5 | 1264766 |
|  |  | #7TS=(Gestational Weight Gain) | 15672 |
|  |  | #8 AB=(Gestational Weight Gain OR Weight Gain, Gestational OR Maternal Weight Gain OR Weight Gain, Maternal OR Pregnancy Weight Gain OR Weight Gain, Pregnancy OR Postpartum Weight Retention OR Weight Retention, Postpartum) | 20874 |
|  |  | #9 #7OR#8 | 25630 |
|  |  | #10 #3AND#AND#9 | 1126 |
| Cochrane Library | 16/6/2025 | #1MeSH descriptor: [Pregnant People] explode all trees | 1037 |
|  |  | #2(Pregnant People OR pregnan OR People, Pregnant OR Pregnant Peoples OR Pregnant Person OR Pregnant Woman OR Woman, Pregnant OR Women, Pregnant OR Pregnant Women):ti,ab,kw | 26929 |
|  |  | #3 #1 OR #2 | 26929 |
|  |  | #4 MeSH descriptor: [Exercise] explode all trees | 40107 |
|  |  | #5(Exercise OR Exercis* OR physical activit* OR exercise training* OR aerobic exercis* OR isometric exercis* OR acute exercis*):ti,ab,kw | 195855 |
|  |  | #6 #4 OR #5 | 199861 |
|  |  | #7 MeSH descriptor: [Weight Gain, Gestational] explode all trees | 255 |
|  |  | #8(Gestational Weight Gain OR Weight Gain, Gestational OR Maternal Weight Gain OR Weight Gain, Maternal OR Pregnancy Weight Gain OR Weight Gain, Pregnancy OR Postpartum Weight Retention OR Weight Retention, Postpartum):ti,ab,kw | 3727 |
|  |  | #9 #7 OR #8 | 3727 |
|  |  | #7 #3 AND #6 AND#9 | 512 |
| Scopus | 17/6/2025 | #1( TITLE-ABS-KEY ( pregnant AND people ) OR TITLE-ABS-KEY ( pregnan* ) OR TITLE-ABS-KEY ( people, AND pregnant ) OR TITLE-ABS-KEY ( pregnant AND peoples ) OR TITLE-ABS-KEY ( pregnant AND person ) OR TITLE-ABS-KEY ( pregnant AND woman ) OR TITLE-ABS-KEY ( woman, AND pregnant ) OR TITLE-ABS-KEY ( women, AND pregnant ) OR TITLE-ABS-KEY ( pregnant AND women ) ) | 1400882 |
|  |  | #2( TITLE-ABS-KEY ( exercise ) OR TITLE-ABS-KEY ( exercis* ) OR TITLE-ABS-KEY ( physical AND activit* ) OR TITLE-ABS-KEY ( exercise AND training* ) OR TITLE-ABS-KEY ( aerobic AND exercis* ) OR TITLE-ABS-KEY ( isometric AND exercis* ) OR TITLE-ABS-KEY ( acute AND exercis* ) ) | 1450019 |
|  |  | #3( TITLE-ABS-KEY ( weight AND gain, AND gestational ) OR TITLE-ABS-KEY ( maternal AND weight AND gain ) OR TITLE-ABS-KEY ( weight AND gain, AND maternal ) OR TITLE-ABS-KEY ( pregnancy AND weight AND gain ) OR TITLE-ABS-KEY ( weight AND gain, AND pregnancy ) OR TITLE-ABS-KEY ( postpartum AND weight AND retention ) OR TITLE-ABS-KEY ( weight AND retention, AND postpartum ) ) | 31206 |
|  |  | #4( TITLE-ABS-KEY ( randomized AND controlled AND trial ) OR TITLE-ABS-KEY ( random* ) OR TITLE-ABS-KEY ( randomized AND controlled AND trial ) OR TITLE-ABS-KEY ( blind ) OR TITLE-ABS-KEY ( double ) ) | 5133940 |
|  |  | #5 #1AND #2 AND #3 AND #4 | 852 |
| Cinahl | 17/6/2025 | S1 SU Pregnant People OR SU pregnan* OR SU pregnan OR SU People, Pregnant OR SU Pregnant Peoples OR SU Pregnant Person OR SU Pregnant Woman OR SU Woman, Pregnant OR SU Women, Pregnant OR SU Pregnant Women | 237964 |
|  |  | S2 (MH “Exercise”) | 62491 |
|  |  | S3 SU Exercise OR SU Exercis* OR SU physical activit* OR SU exercise training* OR SU aerobic exercis* OR SU isometric exercis* OR SU acute exercis* | 209473 |
|  |  | S4 S2 OR S3 | 209473 |
|  |  | S5 SU Weight Gain, Gestational OR SU Maternal Weight Gain OR SU Weight Gain, Maternal OR SU Pregnancy Weight Gain OR SU Weight Gain, Pregnancy OR SU Postpartum Weight Retention OR SU Weight Retention, Postpartum | 1121 |
|  |  | S10(S1 AND S4 AND S9) | 144 |
| PsyciNFO | 17/6/2025 | S1 TI(Pregnant People OR pregnan* OR pregnan OR People, Pregnant OR Pregnant Peoples OR Pregnant Person OR Pregnant Woman OR Woman, Pregnant OR Women, Pregnant OR Pregnant Women) OR AB(Pregnant People OR pregnan* OR pregnan OR People, Pregnant OR Pregnant Peoples OR Pregnant Person OR Pregnant Woman OR Woman, Pregnant OR Women, Pregnant OR Pregnant Women) | 58089 |
|  |  | S2 TI(Exercise OR Exercis* OR physical activit* OR exercise training* OR aerobic exercis* OR isometric exercis* OR acute exercis*) OR AB(Exercise OR Exercis* OR physical activit* OR exercise training* OR aerobic exercis* OR isometric exercis* OR acute exercis*) | 125119 |
|  |  | S3TI(Gestational Weight Gain OR Weight Gain, Gestational OR Maternal Weight Gain OR Weight Gain, Maternal OR Pregnancy Weight Gain OR Weight Gain, Pregnancy OR Postpartum Weight Retention OR Weight Retention, Postpartum) OR AB(Gestational Weight Gain OR Weight Gain, Gestational OR Maternal Weight Gain OR Weight Gain, Maternal OR Pregnancy Weight Gain OR Weight Gain, Pregnancy OR Postpartum Weight Retention OR Weight Retention, Postpartum) | 1004 |
|  |  | S4 S1 AND S2 AND S3 | 167 |
| ProQuest | 17/6/2025 | S1Breast Feeding OR Breast Feeding OR Breast Fed OR Breastfeeding OR Breastfed OR Breast Feeding, Exclusive OR Exclusive Breast Feeding OR Breastfeeding, Exclusive OR Exclusive Breastfeeding | 107488 |
|  |  | S2 Internet OR Remote Consultation OR Text Messaging OR WeChat OR Smartphone | 524159 |
|  |  | S3 randomized controlled trial OR randomized OR placebo OR quasi-experimental studies OR quasi experimental studies OR quasi-experimental study OR studies, quasi-experimental OR study, quasi-experimental | 816330 |
|  |  | S4 S1 AND S2 AND S3 | 32 |
| Wan Fang | 18/6/2025 | 主题：（孕妇 or 孕产妇 or 产妇 or 妇女 or 孕期 or妊娠 or 怀孕）and 主题：（运动 or 训练 or 锻炼 or 有氧运动 or有氧训练 or抗阻力运动）and 主题：(增重过度 or 增重过多 or 体重增长不当 or 体重增加)) | 200 |
| Vip | 18/6/2025 | [(((((((题名或关键词=孕妇 OR 题名或关键词=孕产妇) OR 题名或关键词=产妇) OR 题名或关键词=妇女) OR 题名或关键词=孕期 or妊娠) OR 题名或关键词=怀孕) AND (((题名或关键词=运动 OR 题名或关键词=训练) OR 题名或关键词=锻炼) OR 题名或关键词=有氧运动 or有氧训练 or抗阻力运动)) AND (((题名或关键词=增重过度 OR 题名或关键词=增重过多) OR 题名或关键词=体重增长不当) OR 题名或关键词=体重增加))](https://qikan.cqvip.com/Qikan/search/index?LngMySearHistoryIdGuid=7c59b7ed-d475-48a5-addf-a93a071947bd&from=Qikan_Article_History) | 14 |
| 知网 | 18/6/2025 | (主题:孕妇)OR(篇关摘:孕产妇+产妇+妇女+孕期+娠+怀孕(精确))AND(主题:运动)OR(篇关摘:训练 +锻炼+有氧运动+有氧训练+抗阻力运动(精确))AND(主题:增重过度)OR(篇关摘:增重过多+体重增长不当 + 体重增加(精确)) | 61 |
| CMB | 18/6/2025 | 1) "运动"[常用字段:智能] OR "训练"[常用字段:智能] OR "锻炼"[常用字段:智能] OR "有氧运动"[常用字段:智能] OR "有氧训练"[常用字段:智能] OR "抗阻力运动"[常用字段:智能] | 518801 |
|  |  | 2) "增重过度"[常用字段:智能] OR "增重过多"[常用字段:智能] OR "体重增长不当"[常用字段:智能] OR "体重增加"[常用字段:智能] | 7386 |
|  |  | 3) "孕妇"[常用字段:智能] OR "孕产妇"[常用字段:智能] OR "产妇"[常用字段:智能] OR "妇女"[常用字段:智能] OR "孕期"[常用字段:智能] OR "妊娠"[常用字段:智能] OR "怀孕"[常用字段:智能] | 564655 |
|  |  | 4) (#3) AND (#2) AND (#1) | 189 |

**Supplementary Figure 1: Risk of Bias Assessment** of Included Studies**—Graph for Randomized Controlled Trial (RCT)**

**Supplementary Figure 2: Risk of Bias Assessment** of Included Studies**—Summary for Randomized Controlled Trial (RCT)**

| **Supplementary Table 2.** Results of node-splitting analysis | | | | | | | |
| --- | --- | --- | --- | --- | --- | --- | --- |
| Side | Direct |  | Indirect |  | Difference | |  |
|  | Coef. | Std. Err. | Coef. | Std. Err. | Coef. | Std. Err. | P>z |
| A B * | -3.123255 | 1.282395 | 6.068498 | 3.804343 | -9.191753 | 4.026167 | 0.022 |
| A H | 4.130618 | 1.258216 | -4.031478 | 1.441571 | 8.162095 | 1.920417 | 0.000 |
| B H * | 2.215998 | .791598 | 15.12749 | 4.020588 | -12.91149 | 4.097152 | 0.002 |
| C H * | .4751609 | 1.019579 | .8910131 | 1206.037 | -.4158523 | 1206.037 | 1.000 |
| D H * | 1.59 | 2.267974 | .8224108 | 2823.102 | .7675892 | 2823.103 | 1.000 |
| E H * | -.5270739 | 1.536307 | 1.107105 | 1740.122 | -1.634179 | 1740.122 | 0.999 |
| F G* | 3.51 | 2.574638 | 4.036728 | 17972.14 | -.5267275 | 17972.14 | 1.000 |
| F H | 3.51 | 2.574638 | 4.036728 | 17972.14 | -.5267275 | 17972.14 | 1.000 |
| G H | 3.94 | 2.660034 | 1.827949 | 3371.58 | 2.112051 | 3371.581 | 1.000 |

*Note*. A = Resistance exercise, B = Aerobic exercise, C =Combined Exercise, D = Virtual exercise, E =Supervised Exercise Training, F = Pilates training, G = Childbirth training, H=

Usual care.

| **Supplementary Table 3** Node-splitting results in sensitivity analysis | | | | | | | |
| --- | --- | --- | --- | --- | --- | --- | --- |
| Side | Direct |  | Indirect |  | Difference | |  |
|  | Coef. | Std. Err. | Coef. | Std. Err. | Coef. | Std. Err. | P>z |
| AB * | -5.770001 | .8366558 | -1.07718 | 445.9941 | -4.692821 | 445.9949 | 0.992 |
| BF* | 1.63972 | .5832593 | 6.890046 | 519.7708 | -5.250326 | 519.771 | 0.992 |
| CF* | .4636333 | .5687521 | -8.306067 | 1404.854 | 8.769701 | 1404.856 | 0.995 |
| DF* | 1.59 | 1.051135 | -8.211004 | 2878.56 | 9.801003 | 2878.56 | 0.997 |
| EF* | 1.14 | .8034997 | -8.289444 | 2428.938 | 9.429444 | 2428.938 | 0.997 |

*Note*. A = Resistance exercise, B = Aerobic exercise, C =Combined Exercise, D = Virtual exercise, E =Supervised Exercise Training, F= Usual care

**Supplementary Table 4:Network meta-analysis league table after exclusion of studies at high risk of bias**

| Aerobic exercise | 0.05 (-2.31,2.41) | 0.50 (-1.45,2.45) | 1.18 (-0.37,2.72) | 1.64 (0.50,2.78) | 5.77 (4.13,7.41) |
| --- | --- | --- | --- | --- | --- |
| -0.05 (-2.41,2.31) | Virtual exercise | 0.45 (-2.14,3.04) | 1.13 (-1.22,3.47) | 1.59 (-0.47,3.65) | 5.72 (2.85,8.59) |
| -0.50 (-2.45,1.45) | -0.45 (-3.04,2.14) | Supervised Exercise Training | 0.68 (-1.25,2.61) | 1.14 (-0.43,2.71) | 5.27 (2.73,7.82) |
| -1.18 (-2.72,0.37) | -1.13 (-3.47,1.22) | -0.68 (-2.61,1.25) | Combined Exercise | 0.46 (-0.65,1.58) | 4.59 (2.34,6.85) |
| -1.64 (-2.78,-0.50) | -1.59 (-3.65,0.47) | -1.14 (-2.71,0.43) | -0.46 (-1.58,0.65) | Usual care | 4.13 (2.13,6.13) |
| -5.77 (-7.41,-4.13) | -5.72 (-8.59,-2.85) | -5.27 (-7.82,-2.73) | -4.59 (-6.85,-2.34) | -4.13 (-6.13,-2.13) | Resistance exercise |

**Supplementary Figure 3:Publication bias for** Gestational Weight Gain

Supplementary Figure 4：SUCRA ranking after exclusion of studies at high risk of bias

Supplementary Table S5:GRADE Assessment of the Comparison Between Aerobic Exercise and Usual Care

| Domain | Assessment |
| --- | --- |
| Risk of bias | Serious |
| Inconsistency | Not serious |
| Indirectness | Serious |
| Imprecision | Serious |
| Publication bias | Undetected |
| Overall certainty | Low |
